# Assessing the spatial equity of the aged care institutions based on the improved potential model: a case study in Shanghai, China

**DOI:** 10.3389/fpubh.2024.1428424

**Published:** 2024-08-29

**Authors:** Chenyang Wang, Xiuli Geng

**Affiliations:** ^1^Business School, University of Shanghai for Science and Technology, Shanghai, China; ^2^School of Intelligent Emergency Management, University of Shanghai for Science and Technology, Shanghai, China

**Keywords:** improved potential model, spatial equity, spatial distribution, spatial configuration, aged care institutions

## Abstract

With the spread of an aging society, the demand for aged care institutions among older adults is increasing. The inadequate supply and distribution of aged care institutions have led to an increasing concern about spatial equity in aged care institutions. Most studies have utilized accessibility to assess spatial equity from the supply perspective, while the demand perspective has received little attention. In addition, few studies have evaluated the spatial equity of aged care institutions at grid resolution. Therefore, this study takes Shanghai as an example to analyze aged care institutions from both the supply and demand perspectives. By proposing an improved potential model, at a network resolution of 500 × 500, the spatial equity of aged care institutions is more refined. The results show that aged care institutions and the older population in Shanghai are predominantly concentrated in the downtown area and surrounding regions. However, the results obtained from the Lorenz curve and Gini coefficient indicate the allocation of pension beds based on population size is proportional across different districts of Shanghai. When considering the quality indicators of aged care institutions and introducing the improved potential energy model to calculate spatial accessibility, an imbalance between regions in Shanghai still exists and needs further optimization.

## 1 Introduction

The world is experiencing unprecedented population aging (1). This phenomenon is prevalent in China. According to the results of the seventh population census released by the National Bureau of Statistics of China, 264 million people were 60 years or older as of 2020, accounting for 18.7% of the total population, including 190 million individuals aged 65 years or older (2). Among the aging population, various underlying physiological changes are more likely to occur, increasing the risk of diseases and reducing mobility (3, 4). Institutional care is better or indispensable for the older due to frailty, poor health, and disability. If aged care institutions are appropriately configured based on the needs of the older, more individuals will be able to access essential services in their respective areas. Therefore, the importance of spatial equity in aged care institutions cannot be ignored. Assessing spatial equity can effectively examine the rational allocation and distribution of public facilities and resources within a region and help urban planners better optimize the spatial equity pattern of public facilities. This is theoretically and practically crucial for ensuring the wellbeing of the older, addressing population-aging trends, and achieving social fairness.

As an essential part of public social services, the construction, operation, and maintenance of aged care institutions depend on government supervision. The main goal of the configuration and operation of institutions for the older is equity. Equity refers to the quality of being equal or fair in a situation or distribution, which would affect individual wellbeing (5). Spatial equity in aged care institutions implies ensuring that the older enjoy equal access to older adult care services and that development opportunities are relatively equitable between regions. As a key indicator of spatial equity, spatial accessibility helps to understand whether institutions are available in sufficient numbers and in an equitable manner.

Spatial accessibility reflects the barriers to accessing necessary facilities. Assessing spatial accessibility can effectively identify areas where public facilities are scarce. From the perspective of aged care institutions, accessibility is an important factor in evaluating their service capacity and, thus, the rationality of their spatial location. At the same time, the size, service quality, and geographical location of aged care institutions are also important factors that affect accessibility. The accessibility of aged care institutions can effectively measure the correlation between the older population and aged care institutions.

The models of accessibility commonly used in research include the ratio method (6), the floating catchment area method (7, 8), the minimum distance model (9), potential models (10, 11), and others. Among these models, the ratio method is limited in its ability to consider situations within the study unit, while the minimum distance model is unable to account for resource availability and quantity on the supply side. The two-step floating catchment area method ignores distance attenuation effects, and it is difficult to determine extreme distance or time values. In comparison, potential models can incorporate considerations of both residential demands and spatial barriers, reflecting more precisely the conditions by which residents can access facility resources within smaller areal units under study (12, 13). Additionally, during the 13th Five-Year Plan period, significant improvements were made in the older service system and evaluation system of aged care institutions, leading to higher requirements for their service quality (14). Therefore, it becomes crucial to focus on relevant indicators representing the quality of aged care institutions when assessing their spatial equity.

Although scholars have conducted considerable research on the spatial equity of aged care institutions, there are still some limitations in the previous studies. On the one hand, the common idea is to analyze spatial equity from the perspective of the supply of aged care institutions. It relies on the differences in public facility services between social groups or spatial units to assess the degree of equity (5). This is a useful but incomplete perspective of spatial equity. Equity should not only address the existence of differences between supply and demand but also the satisfaction of residents' needs. The measurement of demand can be expressed by population density, age group system, and population combined with other indicators. Therefore, the assessment of spatial equity should represent a balance between the accessibility calculation of the supply of aged care institutions and the actual demand of residents (15). On the other hand, existing studies applying potential accessibility models typically use counties (16, 17) or neighborhoods (18) as the minimum unit of analysis in examining spatial accessibility within a region. In fact, choosing a smaller unit of study can more effectively capture the variations in spatial accessibility to aged care institutions among residents.

This paper focuses on investigating the spatial equity of aged care institutions in Shanghai, one of the earliest aging cities in China. The study aims to address the existing imbalance and insufficiency in the development of aged care institutions in the city. This study evaluates the spatial equity of aged care institutions in three steps. First, this study analyses the spatial distribution of the older population and aged care institutions from the perspective of supply and demand based on the attribute data of aged care institutions and the older. Then, the spatial differences in the accessibility of aged care institutions are evaluated by combining the spatial location of service recipients (older adults) and their distribution density and considering the spatial barrier between facilities and residents. Finally, the resultant characteristics of the spatial distribution are overlaid with the results of the accessibility evaluation to explore the spatial discrepancy in the service capacity of aged care institutions in the study area. It is then determined whether the spatial distribution of aged care institutions in the study area is equitable, i.e., whether the older enjoy relatively equitable services from aged care institutions in the study area.

The remainder of the paper is organized as follows: Section 2 discusses the literature on spatial equity and spatial accessibility. Section 3 describes the study area, data sources, and data processing. Section 4 presents the improved potential model and the methodology for the Gini coefficient. Section 5 presents the results of the assessment of spatial distribution and spatial accessibility in Shanghai. Section 6 discusses the conclusions and recommendations for future research. Section 7 summarizes the full text and puts forward the outlook.

## 2 Literature review

### 2.1 Spatial equity

Spatial equity comes from the need for layout planning of public facilities, and a reasonable spatial layout can help improve the quality of life of individuals and promote social equity (19). The study of spatial equity in public service facilities has become one of the hot topics in recent years. Spatial equity is crucial to solving the social problems of public facilities and services, including parks and green areas, hospitals, gymnasiums, libraries, and aged care institutions. Based on different research purposes, researchers have different meanings of spatial equity, which can be divided into three categories. One is the consistent treatment of people in the social environment of society as a whole (20, 21). The second is the degree of rationality in considering the layout of facilities based on the different needs of the public and reducing the degree of inequality in the provision of facilities due to class differences (22–24). The third is that spatial equity should ensure that every resident receives the same services and that residents can have a more equal living environment after using public service facilities.

For assessing the spatial equity of resources for older adults, we must consider both whether resource users have equal access to public service resources and whether the distribution of resources enhances overall social welfare without excessive local disparities (25). However, empirically measurable indicators are required to evaluate spatial equity in practical application (26, 27). Evaluating spatial accessibility can effectively identify areas lacking public facilities, providing a quantitative basis for studying spatial equity (28, 29).

### 2.2. Spatial accessibility

Accessibility as a widely accepted concept in urban planning, transportation planning, geography, and various other scientific fields (30, 31) is a valid measure of the equity of spatial configuration of public resources (32). The concept of accessibility is generally defined as a measure of the ease of getting from an origin to an endpoint (29, 33–35).

In previous studies, measures of accessibility are generally divided into three categories, methods based on cumulative opportunities, and methods based on utility, methods based on spatial interactions. The methods based on cumulative opportunities are location-based metrics that assume that people will use the nearest public facility to them and calculate the number of opportunities available within a certain distance, the larger the number, the higher the accessibility. Methods based on this idea include the isocline method and floating catchment area method (36), which are easy to understand relatively simple, and have a wide range of applications. The methods based on utility measures are methods that measure, weigh, combine, evaluate, and determine the priority of potential actions based on a large number of reference factors. These methods require additional transportation, land use, economic, and social data inputs, and research development when performing accessibility analysis (33, 37, 38). The methods based on spatial interactions were first developed by Hansen (39) introduced the physical gravitational model into spatial interaction and started to study it, which mainly calculates the sum of potentials of all facility points in the study area at that point, and the larger the sum of potentials, the higher the accessibility. The methods based on this idea mainly include the potential model (including various improved formulas), the nuclear density method, etc.

When scholars are faced with a multitude of methodological choices, they often customize accessibility methods based on the subjects being studied to yield more accurate and realistic outcomes. For example, Macfarlane et al. (40) believes that in studying the accessibility of public facilities, considering the differences in improvement among different groups of people requires a clear definition of accessibility and measurement methods. Zhang et al. (19) studied the spatial differences in the accessibility of community service resources for the older in China, and they combined four key elements of travel behavior to simulate a more realistic travel behavior of the older population. Kelobonye et al. (41) propose an opportunity accumulation approach that includes assessing the accessibility and spatial equity of urban services in studying the mediocrity of public facilities. Rong et al. (42) studied the spatial equity of hospitals by incorporating population competition and hospital rank factors to obtain a more comprehensive and accurate measure of residents' medical visits. Therefore, when using accessibility for evaluation, incorporating more representative and characteristic indicators of the study subjects can make the results more accurate and reasonable. When assessing the accessibility of aged care institutions, introducing service quality indicators can more accurately evaluate and compare the actual situations of older adult care services in different regions, ensuring the fairness of resource allocation.

Based on the above-mentioned literature review, to examine the spatial equity of aged care institutions in Shanghai, this study mainly considers two aspects. For the first aspect, spatial distribution and service level of aged care institutions are both considered from the perspective of supply and demand. For the second aspect, the improved potential model is proposed to analyze the spatial accessibility of different aged care institutions. Through the analysis of the above results, a holistic evaluation of spatial equity can help urban planners improve the spatial pattern and combat population aging.

## 3 Study area and data sources

### 3.1 Study area

**Figure 1:**
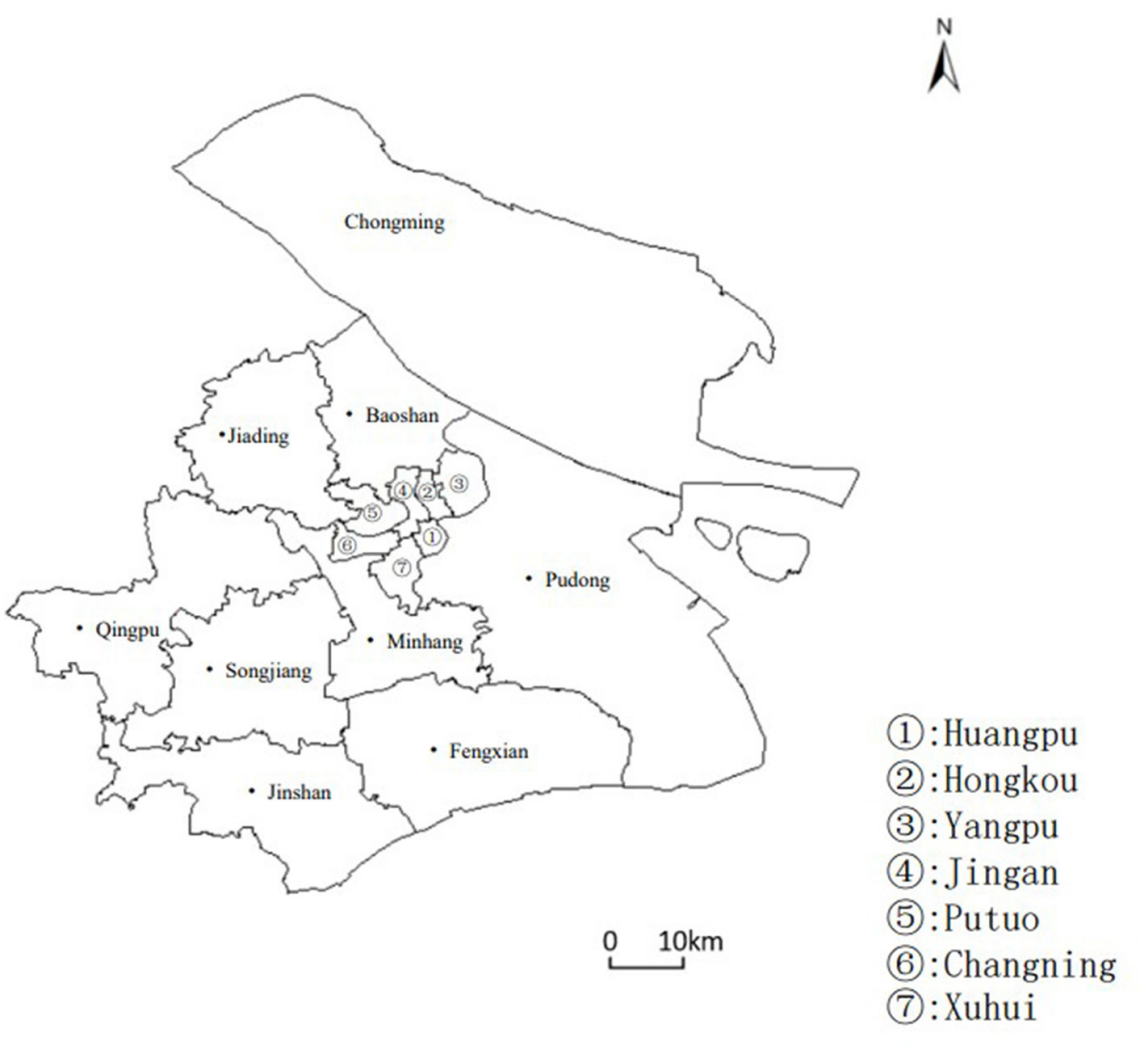
Overview of Shanghai's Administrative Divisions as of 2022. This figure highlights the analysis of the distribution and area of various administrative districts in Shanghai, including the division of the central urban area, specifically covering Huangpu District, Xuhui District, Changning District, Jing'an District, Putuo District, Hongkou District, and Yangpu District, with the remaining districts being classified as suburban areas.

**Figure 2:**
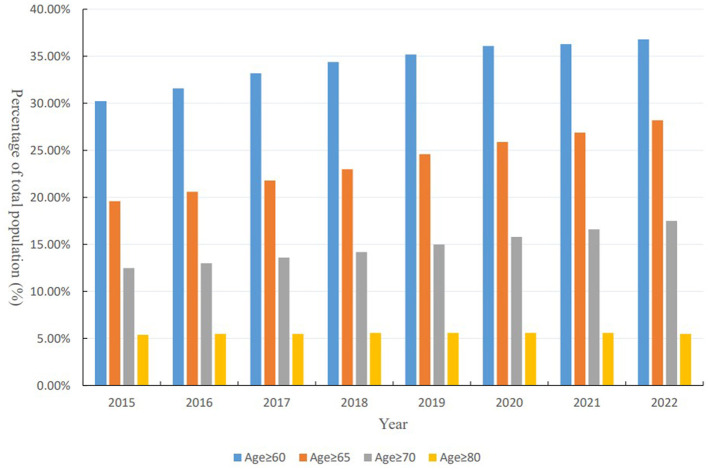
Annual changes in the percentage of Shanghai's aging population (2015–2022). This figure presents the percentage of the total population in Shanghai that falls into different age brackets−60 years and above, 65 years and above, 70 years and above, and 80 years and above—from 2015 to 2022.

Shanghai, located on China's eastern coast at the mouth of the Yangtze River and facing the Pacific Ocean, stands out as one of China's most vibrant and open areas for economic development. As of the end of 2021, Shanghai's administrative division spans 6,340.5 square kilometers, comprising 16 districts, 107 streets, 106 towns, and two townships, with a permanent population of 24,894,300 predominantly located in the central urban zone (Figure 1). Shanghai is the first city in China to enter an aging society and has the highest degree of population aging in the country. In recent years, the older population has continued to expand, with the proportion of older adults in the total population increasing annually, as shown in Figure 2. As of 2022, the number of older adults aged 60 and above in the city's permanent population was 5.5366 million, accounting for 36.8% of the total population. The number of older adults aged 80 and above was 831,500, representing 15.0% of the older population aged 60 and above and 5.5% of the total population. Over 1.7 million older adults living alone or in households consisting solely of older adults need care, a number expected to grow during the “14th Five-Year Plan” period. The aging population, coupled with trends such as low birth rates, a high proportion of the older, smaller family sizes, and households consisting solely of older adults, together constitute the complex aging demographic pattern of Shanghai. According to the Shanghai municipal government's plan, by 2035, the city's permanent population is expected to stabilize at 25 million. Meanwhile, the pace of population aging will continue to accelerate, leading to a growing demand for older adult care services. This trend presents both opportunities and challenges for Shanghai, making the identification of spatial equity in aged care institutions significantly valuable and important.

### 3.2 Data on aged care institutions

According to the “Regulations of Shanghai Municipality on Older Adult Care Services,” older adult care service institutions refer to facilities that provide full-time centralized accommodation and care services for the older, as well as home and community-based older adult care service institutions that offer home and community older adult care services. Among them, aged care institutions primarily serve as public service facilities for institutional older adult care planned by the government. Therefore, this study focuses on aged care institutions as the research subject. The attributes used to evaluate the equity of senior care institutions in this study are primarily divided into two categories: basic information (such as facility name, location, and size) and geographic coordinates. To begin with, basic information on senior care institutions is obtained through the integration of data gathered through Python web scraping techniques from the Shanghai Senior Care Service Platform, as well as summary information of Shanghai senior care institutions released by the Shanghai Municipal Civil Affairs Bureau. Subsequently, geographic coordinate information of aged care institutions is acquired through the utilization of AMAP's API for developers and programming in Python. Finally, dataset integration is performed using the names of both datasets to correlate and eliminate duplicated entries, resulting in the dataset used for analysis in this article. The descriptive information on the aged care institutions is shown in Table 1.

**Table 1:** Partial data of aged care institutions in different administrative districts.

**Administrative area**	**Number of beds**	**Number of aged care institutions**	**Number of graded aged care institutions**
Pudong	25,874	133	86
Huangpu	2,935	21	12
Xuhui	6,130	41	28
Changning	5,892	35	12
Jingan	4,475	30	19
Putuo	6,645	45	27
Hongkou	7,024	35	23
Yangpu	9,908	58	31
Minhang	12,575	50	30
Baoshan	11,735	46	23
Jiading	10,279	28	21
Jinshan	7,723	30	25
Songjiang	7,290	22	16
Qingpu	6,282	19	4
Fengxian	5,419	27	19
Chongming	6,533	38	19

### 3.3. Data on the residential population

**Figure 3:**
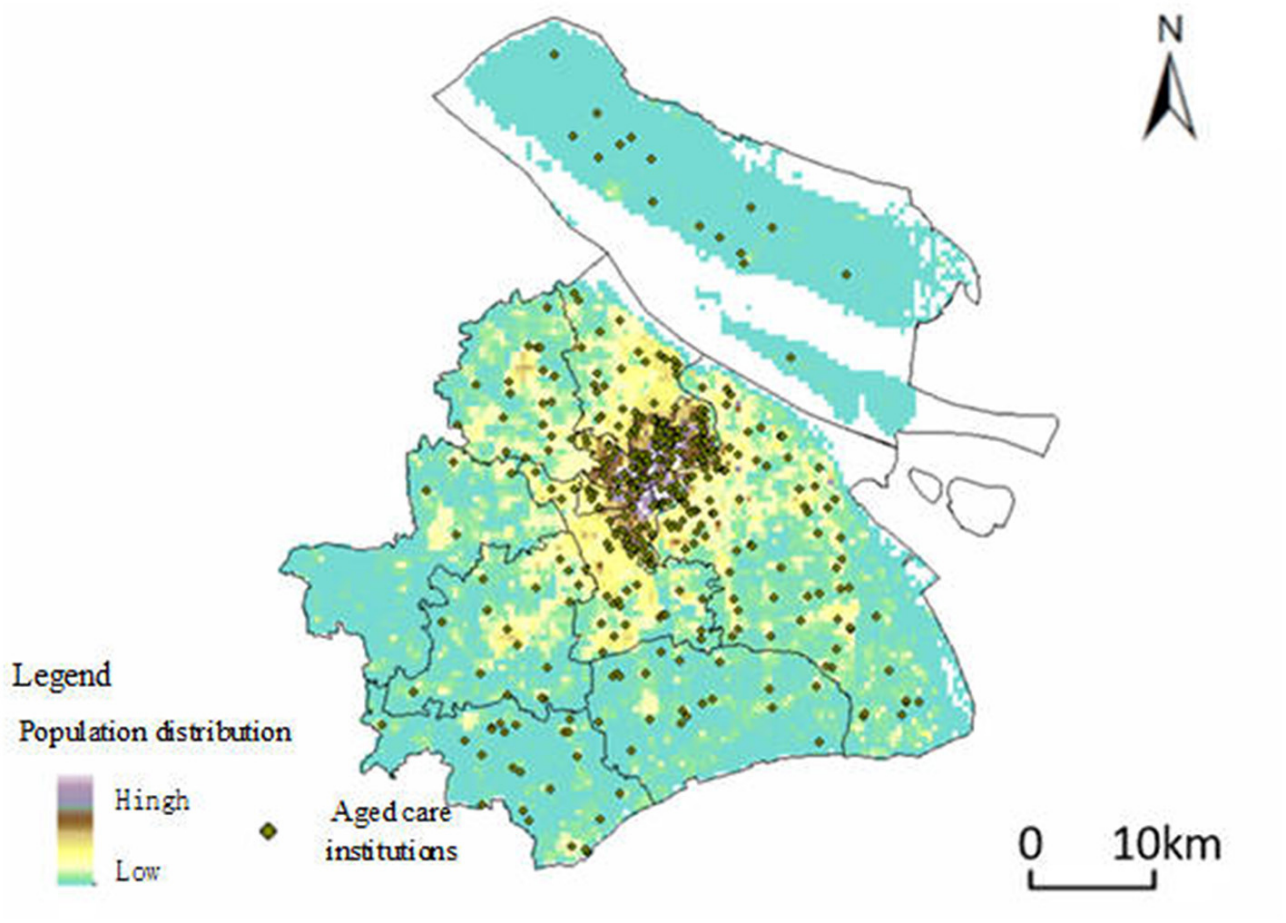
Spatial distribution of Aged Care Institutions and population density of older adults in Shanghai. This figure depicts the spatial distribution of aged care institutions in Shanghai, represented by green dots, overlaid on a heatmap indicating the population density of residents aged 60 and above. The heatmap uses a color gradient, where areas with a higher concentration of older adults are shown in darker shades, and areas with lower concentrations are in lighter shades.

The accessibility of spatially distributed aged care institutions is influenced by multiple factors including the characteristics of the aged care institutions (such as their level or tier, number of beds, and location), as well as the potential demand for services from the population. To enhance the reliability of our research findings, this study obtained data on the distribution of the population over 60 years old in China through WorldPop (https://www.worldpop.org/). Furthermore, this paper corrected the population figures of each administrative district in Shanghai using the Shanghai Statistical Yearbook 2022 to obtain a more accurate distribution of the older population in Shanghai (shown in Figure 3).

## 4 Methodology

### 4.1 The improved potential model

A potential model is a well-established approach in regional economics and geography, which draws inspiration from the law of gravity in physics to analyze social and economic spatial interactions. The model can indicate the supply status and spatial decay factors of urban infrastructure in a specific geographical space. The potential model is provided in Equation 1:


(1) $$\begin{array}{l} A_{i}=\overset{n}{\underset{j=1}{\sum }}A_{ij}=\overset{n}{\underset{j=1}{\sum }}\frac{M_{j}}{D_{ij}^{\beta }} \end{array}$$

where *A*_*ij*_ denotes the potential generated by aged care institutions *j* to residential point *i*. *M*_*j*_denotes the service capacity that aged care institutions can provide, and *D*_*ij*_ denotes the travel impedance between residential point *i* and aged care institutions *j. A*_*i*_ denotes the sum of the spatial accessibility of residential point *i* to aged care institutions within the study area. β denotes the travel friction coefficient. For different public facilities, the value of β will affect the accuracy of the calculation results.

Based on the basic potential model Equation 1, Weibull (43) introduced the population size factor *V*_*j*_ in 1976 to further improve the potential model. The basic formula is provided in Equation 2:


(2) $$\begin{array}{l} A_{i}=\overset{n}{\underset{j=1}{\sum }}\frac{M_{j}}{D_{ij}^{\beta }V_{j}}\;,\;V_{j}=\overset{m}{\underset{k=1}{\sum }}\frac{P_{k}}{D_{kj}^{\beta }} \end{array}$$

where *m* and *n* represent the number of residential areas and the number of aged care institutions. *P*_*k*_ denotes the population of residential point *k*; *V*_*j*_ is the impact of population size; $D_{kj}^{\beta }$ represents the impedance between residential point *k* and facility point j on the premise that the friction coefficient is β. Although the introduction of population size can solve the problem of resource competition caused by residents living in the same aged care institution, it ignores the particularity of aged care institutions.

On the one hand, according to the National Standards of China on Classification and Evaluation of Aged care Institutions, the scale and services of each aged care institution are defined in terms of facilities, operation and management, and services. The higher the level of an aged care institution, the better the evaluation results in all aspects and the greater the attractiveness to the older. When considering the accessibility of aged care institutions, the level of aged care institutions should also be considered for its influence on the older. On the other hand, according to the 14th Five-Year Plan for the Development of Older adult care Services in Shanghai (44), aged care institutions with more than 150 beds are all equipped with medical institutions. The more beds an aged care institution has, the more attractive it is to the older because it is relatively perfect and friendly for the older. Based on the issues discussed in this paper, we set up a multi-level service radius *d* to constrain the service scale of aged care institutions and introduce the influence coefficient *S*_*ij*_ to reflect the influence of the level and scale of aged care institutions on the older. The improved potential model is provided in Equation 3:


(3) $$\begin{array}{c} A_{i}=\sum_{j=1}^{n}\frac{S_{ij}M_{j}}{D_{ij}^{\beta }},\;V_{j}=\sum_{k=1}^{m}\frac{S_{kj}P_{k}}{D_{kj}^{\beta }},\;S_{ij}=1-{\left(\frac{D_{ij}}{d+D_{j}}\right)}^{\beta },\\ d=\{\begin{array}{c} 2h\\ 1h \end{array}\;\frac{M_{j}>500}{M_{j}<500} \end{array}$$

Among them, *S*_*ij*_ represents the influence coefficient of the level and scale of aged care institution *j* on the older distribution area *i*. *S*_*kj*_ represents the influence of the level and scale of aged care institution point *j* on the medical treatment behavior of older adults in distribution area *k*. *D*_*j*_represents different levels of pension institutions represented by the travel restrictions. *D*_*ij*_ represents the time cost based on the distance of the road network between the older adult distribution area *i* and aged care institution point *j*. *M*_*j*_ denotes the number of beds that can be provided by the aged care institutions. The “15-min living circle” concept proposed by the Ministry of Shanghai Municipal Government and other research findings were taken into account to set travel time limits for different levels of senior living facilities. Fifteen minutes set for aged care institutions below Level 3, 0.5 h for Level 3, 1 h for Level 4, and 2 h for Level 5. Accessibility is measured in terms of the number of beds per older adult. To conform to the format of the Shanghai “1,000 people index,” the results will be multiplied by 1,000 to facilitate comparison. The results are multiplied by 1,000 for ease of comparison.

The friction coefficient β is a crucial parameter in the measurement of accessibility. The value varies for different research areas and objects. According to current research, it is found that β is usually more reasonable between 1 and 2 (45–47). When studying the medical field, taking β as 2 can better reveal the differences in spatial accessibility (42, 47). Considering the particularity of the services provided by the aged care institutions, the frequency of the older population obtaining services from the aged care institutions is significantly lower than that of medical facilities, so the friction coefficient β in this paper is taken as 1.

### 4.2. Method of Gini coefficient

This paper utilizes the Lorenz curve and Gini coefficient to assess the fairness of the distribution of urban aged care institutions across administrative districts. The Lorenz curve represents the cumulative percentage of the population against the cumulative percentage of bed occupancy, organized by the number of beds per institution in each district. Each point on the curve indicates the combined resources utilized by a specific percentage of the older population. The Gini coefficient, derived from the Lorenz curve, quantifies the level of equity in resource allocation. It is computed as the ratio of the area between the Lorenz curve and the 45-degree line to the area of maximum inequality. A Gini coefficient close to 0 indicates an equitable distribution and close to 1 indicates an inequitable distribution. The formula for calculating the Gini coefficient is provided in Equation 4:


(4) $$\begin{array}{l} G=1-\overset{n}{\underset{i=1}{\sum }}\left(P_{k}-P_{k-1}\right)\left(T_{k}+T_{k-1}\right) \end{array}$$

where *P*_*k*_ is the cumulative rate of demand for the older, *T*_*k*_ represents the cumulative ratio of the supply of beds in aged care institutions. The Gini coefficient values range from 0 to 1, according to the international standard of Gini coefficient classification, income below 0.2 is regarded as absolute average, 0.2–0.3 as relatively average, 0.3–0.4 as relatively reasonable, 0.4–0.5 as large gap, and when the Gini coefficient reaches 0.5 or above, it indicates a wide gap.

## 5 Results

### 5.1 The results of the distribution of aged care institutions and the older

**Figure 4:**
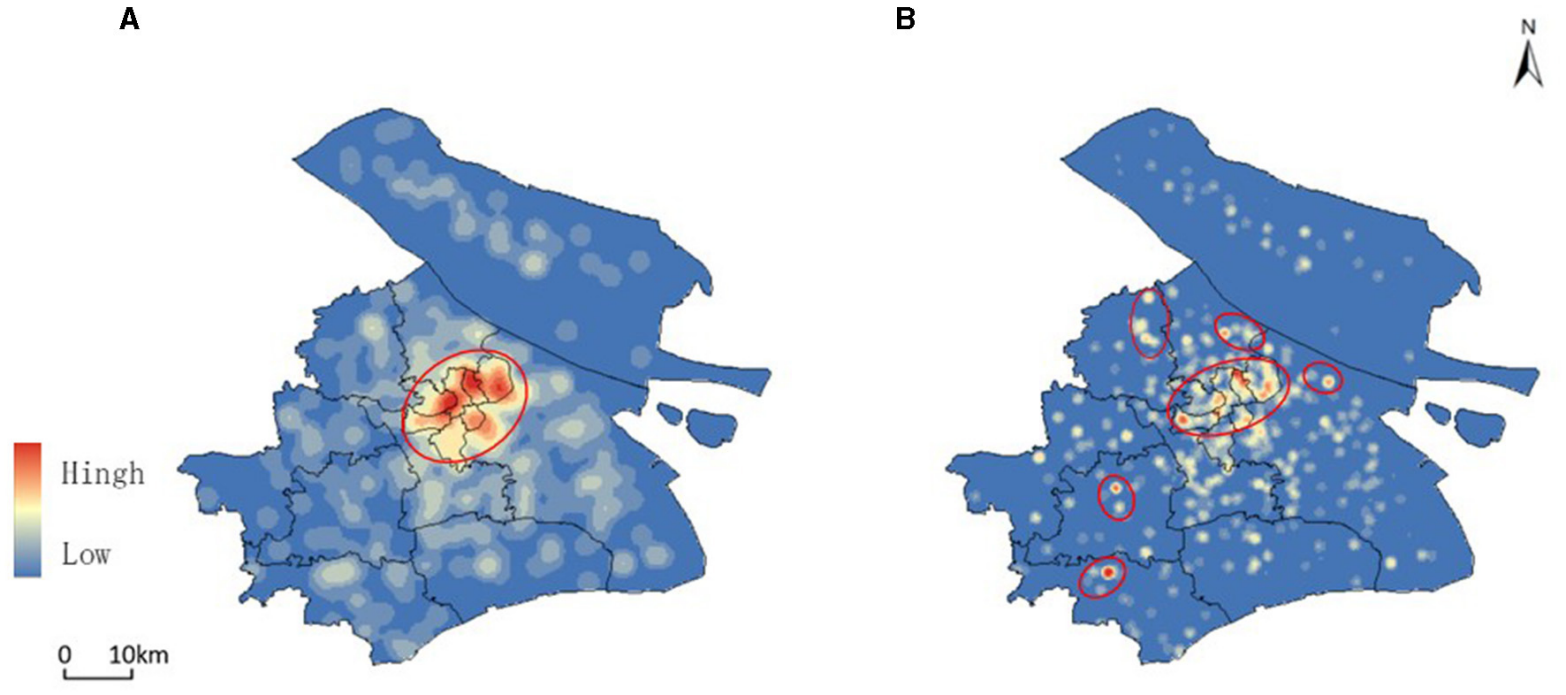
Kernel density analysis of Aged Care Institutions and bed availability in Shanghai (2022). **(A)** shows the kernel density analysis of the distribution of aged care institutions across Shanghai, highlighting areas with a higher concentration of facilities, particularly in the city center. **(B)** illustrates the kernel density of the number of beds available in these institutions, indicating that while the city center has a high density of beds, the distribution is less concentrated compared to the facilities themselves.

According to the data of the Shanghai Older Care Service Platform, as of 2022, there are about 658 aged care institutions in Shanghai, of which Pudong has the largest number of aged care institutions, reaching 133. The top five districts in terms of proportion are Pudong, Yangpu, Minhang, Baoshan, and Putuo, accounting for 20.2%, 8.81%, 7.60%, 7.0%, and 6.84% respectively. Figure 4 displays the results of kernel density analysis for both the number of aged care institutions (Figure 4A) and the number of beds in these institutions (Figure 4B). The city center exhibits more facilities with higher density, while the suburbs have a more scattered distribution. Although the city center has a high density of beds, the distribution is less concentrated compared to the distribution of aged care institutions. Notably, Songjiang, Jinshan, and Jiading districts show higher density areas (marked by red dotted circles).

**Figure 5:**
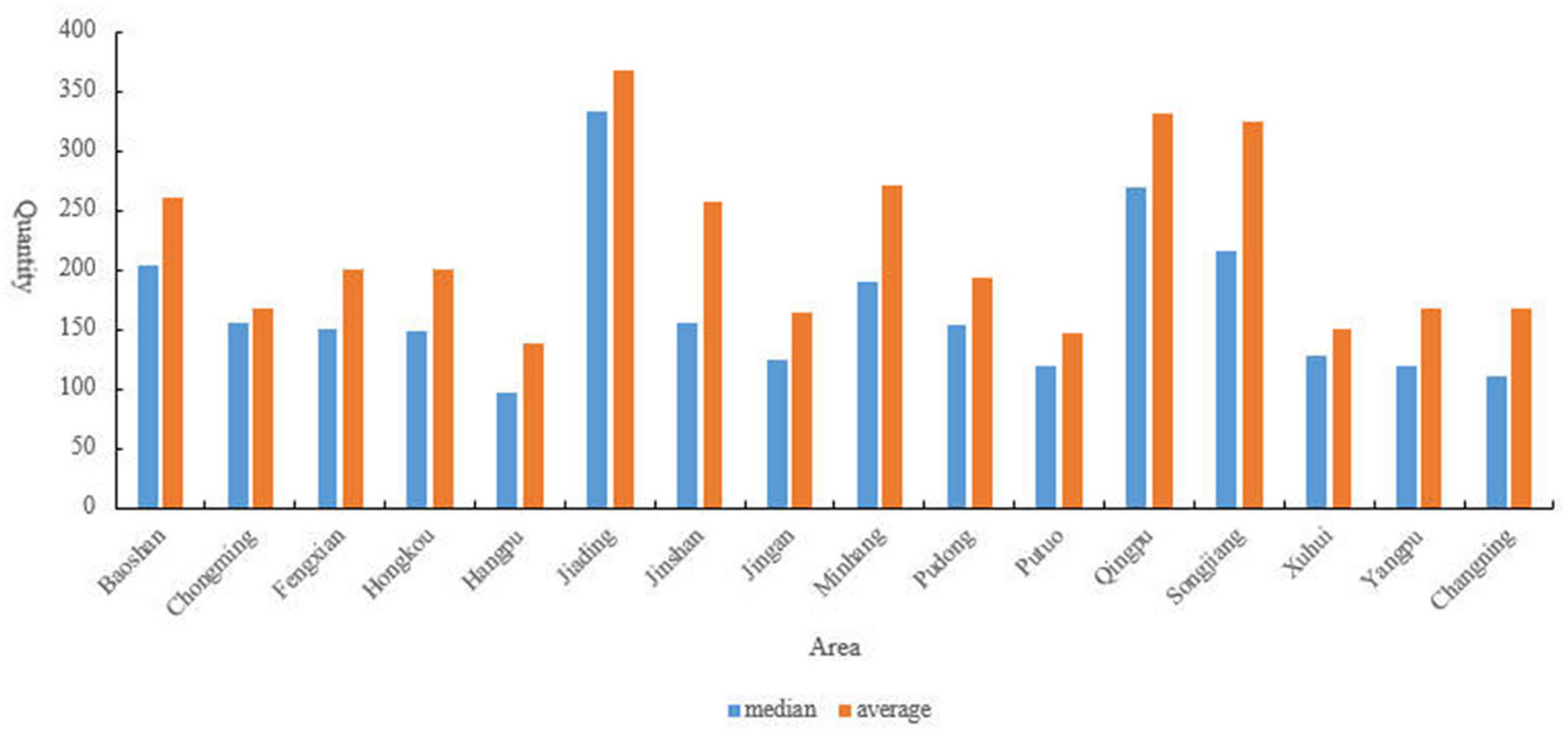
Distribution of median and average scale of aged care institutions across Shanghai's Administrative Regions. This figure presents the median and average number of beds per aged care institution across different administrative regions in Shanghai.

According to the collected data, further statistics were made on the distribution of the scale of aged care institutions in each administrative region. From the perspective of the average scale of facilities (Figure 5), the scale of aged care institutions in the central urban areas is relatively small, with a median of about 150 beds, while the scale of facilities in suburban counties is larger. At the same time, there are large differences in the scale of aged care institutions among the suburbs and counties of each city. Among them, Songjiang, Qingpu, and Jiading have the largest average facility scale, exceeding 300 beds.

**Figure 6:**
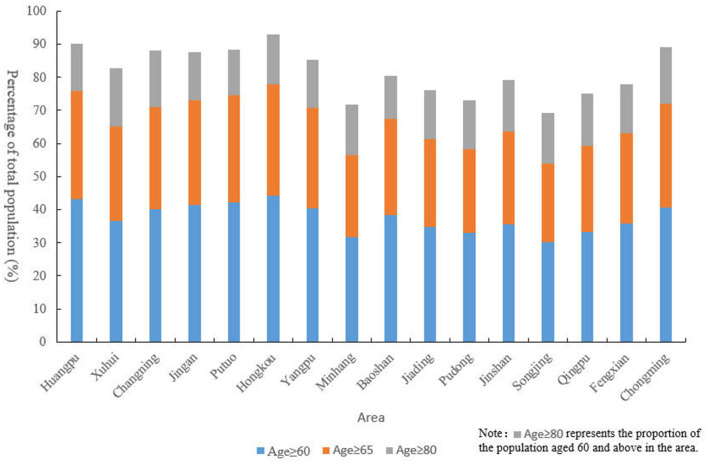
Proportion of older population by age group across Shanghai's Administrative Districts (2022). This figure displays the percentage of the older population in different age groups (60 years and above, 65 years and above, and 80 years and above) across various administrative districts in Shanghai as of 2022.

According to the Shanghai Statistical Yearbook 2022, as of 2022, Pudong is the district with the largest older population, numbering 1.0806 million. The top five districts in Shanghai in terms of older population are Pudong, Yangpu, Baoshan, Minhang, and Putuo, with populations of 1.0806 million, 419,800, 411,000, 402,400, and 380,000 respectively. According to the data shown in Figure 6, based on the degree of population aging, the districts in Shanghai can be categorized as follows: districts with a proportion of the older population (aged 60 and above) exceeding 40% include Huangpu, Jing'an, Putuo, Hongkou, Changning, Yangpu, and Chongming; districts with a proportion of the older population between 35 and 40% include Xuhui, Baoshan, Fengxian, and Jinshan; districts with a proportion of the older population below 35% include Jiading, Songjiang, Qingpu, and Minhang. Among these districts, the top three with the highest proportion of the older population aged 80 and above are Chongming, Changning, and Xuhui. The level of population aging in Shanghai is higher than the national average, with the older population mainly concentrated in the central urban areas. Overall, Shanghai's aged care institutions and the number of older adults have achieved spatial coupling at the city level.

**Figure 7:**
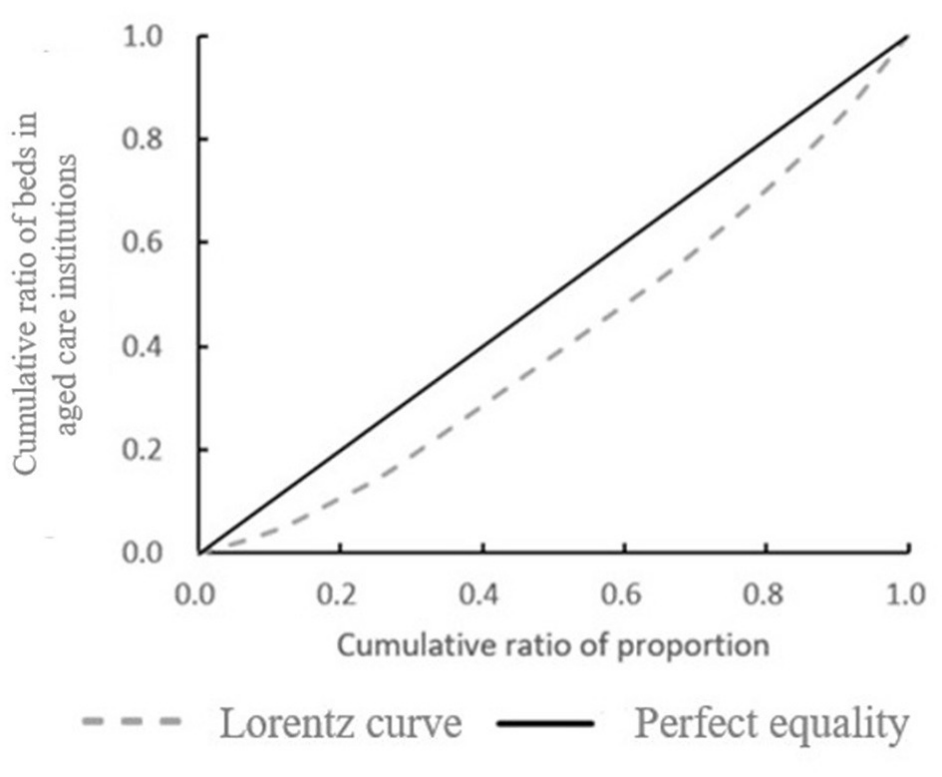
lorenz curve of bed distribution in Aged Care Institutions in Shanghai's Municipal Districts. This figure illustrates the Lorenz curve, depicting the cumulative ratio of beds in aged care institutions against the cumulative ratio of the older population across different municipal districts in Shanghai. The dashed line represents the actual distribution of beds (Lorenz curve), while the solid line represents perfect equality.

According to the data in Table 1, the cumulative proportion of each municipal district is calculated by the ratio of the total number of beds in aged care institutions in the region to the total number of beds in aged care institutions in the region. On this basis, the Lorenz curve was plotted to calculate the cumulative composition ratio of the population in each spatial unit (Figure 7). The Gini coefficient for the distribution of the older population in the jurisdiction was calculated from the Lorenz curve.

According to the distribution of the older population in the study area of the article, based on Equation 4, the Gini coefficient is 0.19. According to the evaluation criteria of the Gini coefficient, the number of beds in aged care institutions in each district within Shanghai can be said to be equitable in terms of population distribution. The number of aged care institution beds in each district is also fair in terms of population distribution. The number of aged care institution beds is positively correlated with the number of the older population. This indicates that there is not much difference in the number of beds in aged care institutions in various districts in Shanghai, and the Shanghai Municipal Government can do a good job in the balanced development of aged care institutions in various regions.

### 5.2 Evaluation results of spatial accessibility

**Figure 8:**
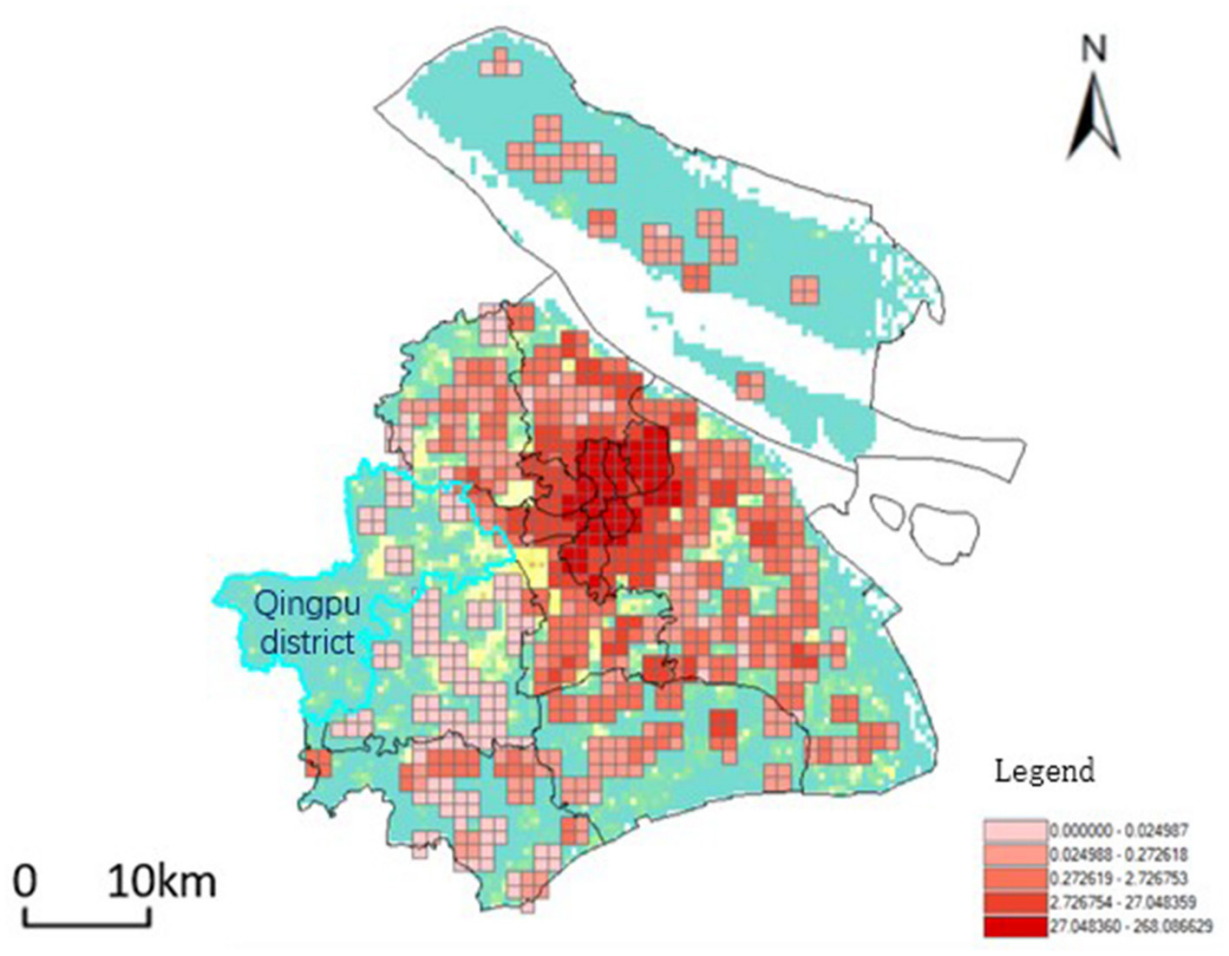
Spatial distribution of accessibility to Aged Care Institutions in Shanghai. This figure presents the interpolated spatial distribution of accessibility values for aged care institutions across Shanghai. The color gradient, with darker shades indicating higher accessibility values, highlights areas with better access to aged care services.

The accessibility values are obtained according to Equation 3. Based on the results of accessibility, the spatial distribution interpolation of aged care institutions is shown in Figure 8. The darker the color, the higher the accessibility value it represents, and the better the accessibility. The spatial accessibility results show that the accessibility of aged care institutions is concentrated in and around a few administrative districts in the city center, and there are some differences and imbalances in aged care institutions and services within each administrative district.

Based on a study of aged care institutions in each district of Shanghai, it was observed that Qingpu District has relatively lower accessibility levels compared to other areas. The presence of the sizable Dianshan Lake and its surrounding water system indicates that the complex topography could affect the overall planning layout of the region, which may hinder the distribution of residential areas and aged care institutions, thereby affecting the accessibility of the entire area.

**Figure 9:**
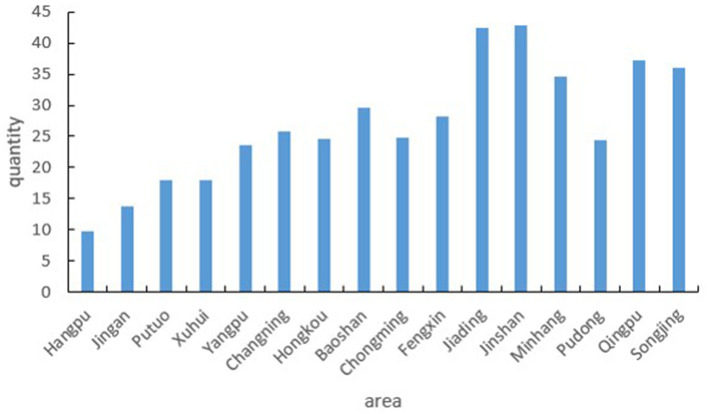
Number of beds in aged care institutions per 1,000 older adults across Shanghai's Administrative Districts. This figure shows the distribution of bed resources in aged care institutions relative to the population of older adults (aged 60 and above) in each administrative district of Shanghai.

Combined with the population data, the number of beds in aged care institutions for every 1,000 older adults in each district. The results show that there are large differences in the distribution of per capita bed resources in different districts (Figure 9). The size and number of aged care institutions in the study area in the city center are much larger than those in the suburbs, while higher-rated institutions are densely distributed, leading to an over-concentration of older adult care resources. The overall area of the downtown is smaller than the area of the suburbs, and the population is larger than the population of the suburbs, with comprehensive accessibility significantly higher than the average for the entire region. The size and number of aged care institutions in the suburbs are significantly smaller than those in the urban areas, and the population size is relatively smaller, but the supply-to-demand ratio is minimal. It is difficult for senior care institutions in the suburbs to cover the real area, and the accessibility level of senior care is significantly lower than that of the whole region.

## 6 Discussion

According to the Shanghai Municipal “14th Five-Year Plan,” continued and profound changes in the size and structure of the population will continue to profoundly affect the pattern of economic and social development in Shanghai. It is expected that during the “14th Five-Year Plan” period, the trend of population aging in Shanghai will become more obvious. At the same time, the problem of imbalance between urban and rural aged-care institutions remains prominent, with “one bed hard to find” in the central urban area coexisting with some unused ones in the suburbs, and a large gap between the construction of aged-care service teams and the demand for aged-care services.

According to the results of this study, although the distribution of the older population does not match the allocation of aged care institutions, the Gini coefficient shows that the number of beds in aged care institutions is roughly proportionate to population size, with little variation across administrative districts. Regarding the spatial accessibility of aged care institutions, the results which take into account level and scale when calculating accessibility demonstrate that accessibility is concentrated in central districts and surrounding areas, while intra-district differences and imbalances in aged care institutions and services persist. Further improvements are warranted.

Therefore, there is still much room for improvement in the planning of aged care institutions and services in Shanghai. Take relevant measures to remedy resource-scarce areas and improve the overall balance. Specific suggestions are as follows:

- Optimizing the spatial layout of aged care institutions: based on the distribution of the older population and population structure, adjust and optimize the spatial layout of institutional older adult care services to enhance the spatial matching degree of supply and demand for older adult care beds. Increase efforts to coordinate the construction and allocation of older adult care beds citywide, promote the flow and selection of beds across districts, and improve the efficiency of bed utilization.
- Enhancing existing aged care institutions: encourage the renovation and optimization of existing aged care institutions, where conditions permit, to gradually increase the proportion of nursing care beds and beds for cognitive impairment care. This aims to fully meet the needs of older individuals with disabilities (including those with dementia) for institutional care. Additionally, improve the service quality of aged care institutions by upgrading and renovating existing facilities to raise their standards. Close or transfer institutions that do not meet the standards to narrow the gap between aged care institutions in central urban areas and suburban areas, thereby alleviating the pressure on central urban areas.
- Optimizing infrastructure reserves: local government departments can optimize existing infrastructure reserves by repurposing some hotels and office buildings to increase the capacity of aged care institutions in areas with concentrated aging populations. Furthermore, a comprehensive approach should be adopted to plan dedicated aged care institutions, selecting locations near medical facilities, parks, and local communities. By strategically positioning these institutions, accessibility, and convenience for residents and medical staff can be improved.

## 7 Conclusion

The increasing aging of the population makes it a pressing issue to eliminate disparities in access to institutional services for the older. It has become important to rationally assess the spatial equity of aged care institutions. This study comprehensively evaluates the spatial equity of aged care institutions in Shanghai. Firstly, the distribution of aged care institutions and the older in Shanghai is analyzed through the collected relevant data. Then the differences in the number of beds for older adults in each sub-district are assessed using the Lorenz curve and the Gini coefficient. Finally, the spatial distribution of older services is determined by introducing quality indicators of aged care institutions and improving the potential energy model for accessibility analysis.

The results of the kernel density analysis show that the city center of Shanghai has more aged care institutions with high density, while the suburbs are relatively dispersed. On this basis, the Gini coefficient is 0.19 based on the population distribution of the study area districts. The distribution of aged care institutions in Shanghai aligns with the population distribution of Shanghai. The accessibility results calculated with the improved potential model show that the accessibility results of the city center are higher than the average, which needs to be further optimized.

According to the results of the study, it is imperative to enhance investment in aged care institutions for future management, taking into account the planning and design of such facilities in the study area. Efforts should be made to augment the quality and capacity of these institutions. Furthermore, a well-balanced distribution of older adult care resources should be pursued to address the issue of regional supply and demand discrepancies. Proactive measures need to be taken to address population aging.

The limitation of this study is the lack of a fine-grained segmentation of the older population. Healthy and ill older adults have different preferences for aged care institutions, and their different choices may lead to different measurable outcomes. Future studies will address these issues.

## Data Availability

Publicly available datasets were analyzed in this study. This data can be found at: https://shyl.mzj.sh.gov.cn/homePage, https://hub.worldpop.org/, and https://tjj.sh.gov.cn/.
